# Nitrates of Synthetic Cellulose

**DOI:** 10.3390/polym18010010

**Published:** 2025-12-19

**Authors:** Vera V. Budaeva, Anna A. Korchagina, Yulia A. Gismatulina, Ekaterina I. Kashcheyeva, Polina A. Gorbatova, Galina F. Mironova, Vladimir N. Zolotukhin, Nikolay V. Bychin, Inna V. Lyukhanova, Lyudmila A. Aleshina, Gennady V. Sakovich

**Affiliations:** 1 Bioconversion Laboratory, Institute for Problems of Chemical and Energetic Technologies, Siberian Branch of the Russian Academy of Sciences (IPCET SB RAS), Biysk 659322, Russia; yakusheva89_21.ru@mail.ru (A.A.K.); julja.gismatulina@rambler.ru (Y.A.G.); massl@mail.ru (E.I.K.); 1402plngorbatova@mail.ru (P.A.G.); yur_galina@mail.ru (G.F.M.); zolo-i@mail.ru (V.N.Z.); eas_i@mail.ru (G.V.S.); 2 Department of Biotechnology, Biysk Technological Institute, Polzunov Altai State Technical University, Biysk 659305, Russia; 3 Laboratory of Materials Science of Mineral Raw Materials, Institute for Problems of Chemical and Energetic Technologies, Siberian Branch of the Russian Academy of Sciences (IPCET SB RAS), Biysk 659322, Russia; nbych@yandex.ru; 4 Radiation Monitoring Sector, Petrozavodsk State University (PetrSU), Petrozavodsk 185910, Russia; luhanova@yandex.ru; 5 Department of Solid-State Physics, Petrozavodsk State University (PetrSU), Petrozavodsk 185910, Russia; alkftt@mail.ru

**Keywords:** synthetic cellulose, nitration, synthetic cellulose nitrate, SEM, FT-IR spectroscopy, X-ray diffraction, TGA/DTA

## Abstract

To avoid dependence on conventional raw materials, global emphasis has been placed on obtaining alternative plant celluloses and the chemical synthesis of cellulose. The use of synthetically derived cellulose as a precursor for cellulose nitrates (NCs) is currently absent in global practice, which underscores the undoubted relevance of this research. Cellulose nitrate (NC) was synthesized in a 138% actual yield by nitration of synthetic cellulose (SC)—a new type of cellulose—prepared by electropolymerization from an aqueous glucose solution in the presence of catalytic tungsten–vanadium heteropolyacid of the 1–12 series with the chemical formula H_6_[PW_10_V_2_O_40_]: a nitrogen content of 11.83%, a viscosity of 198 mPa·s, a high solubility of 91% in an alcohol–ether solvent, and an ash content of 0.05%. SEM provided a general concept of the morphological structure of SC and SC-derived NC. The initial SC consisted of flat, curly fibers with a smooth surface approximately 10–20 μm wide, with no aggregation observed. The fibers of SC-derived NC had a cylindrical shape with a diameter of up to 25 μm and a rough surface. FT-IR spectroscopy revealed that SC and SC-derived NC have the main functional groups characteristic of classical cellulose (3346, 2901, 1644, 1429, 1162, and 1112 cm^−1^) and nitrate esters of cellulose (1650, 1278, 832, 747, and 689 cm^−1^), respectively. For the first time, a full-profile analysis discovered that SC is made up of the monoclinic phase of cellulose Iβ with an antiparallel chain arrangement. SC with a crystallinity index (CrI) of 81–86% was shown to undergo amorphization upon nitration, with the CrI declining to 17% and the crystallite sizes decreasing from 44 × 62 × 59 × 94 Å to 29 × 62 × 26 × 38 Å. Coupled TGA/DTA revealed that SC exhibits a high-temperature endothermic peak of decomposition of 374 °C, with a weight loss of 84%. The thermostable SC-derived NC exhibits a high onset temperature of intense decomposition of 200 °C and an exothermic peak of 208 °C, with a weight loss of 88%, and is characterized by a high specific heat of decomposition of 7.74 kJ/g. This study provides new insights into the functionalization of SC with a high degree of polymerization, expanding the classical concepts of cellulose nitration.

## 1. Introduction

One of the most demanded energy-rich polymers derived from cellulose is cellulose nitrates (NCs), which were first discovered by Henri Braconnot in 1832 and improved by Christian Schönbein in 1846 [1]. Due to their unique characteristics, most notably their nitrogen content, which determines the properties and further application areas, the use of NCs has reached a new level in recent decades, from rocket propellants to biomedicine [2,3]. NCs are leaders in the manufacture of ammunition and in rocket technology [4,5,6], and are used in the mining industry [7] and new composites [8]. Technologies are being developed to produce nanoscale NCs for use in explosive formulations [9].

Because of their low toxicity [10], low-esterified NCs (with a nitrogen content of up to 12.5%) are utilized in dental medicine and cosmetology [11,12], analytical medicine [13,14,15], immunoassays [16,17], in the manufacture of anti-COVID masks [18], and as membrane filters for the detection of bacteria trapped in air conditioners [19], and for the purification of drinking water [20].

Regardless of the field of application, the synthesis of NC is carried out by nitrating cellulose with nitrating mixtures. The mechanism of the highly exothermic esterification reaction involves the substitution of the hydrogen atom of the hydroxyl group (–H–OH) in cellulose by the nitro group (–NO_2_) [3,4,5,6,9,10,13]. By varying the composition and ratio of the nitrating mixture, as well as the conditions of the nitration and stabilization processes, it is possible to obtain NC with tailored characteristics that determine its fields of application [2,21,22].

The conventional feedstocks for the synthesis of NCs are cotton-based [22,23,24,25], wood-based [22,26] and flax-based celluloses [27]. However, a particular emphasis is currently placed on alternative precursors of NCs in order to avoid dependence on conventional feedstocks. It has been demonstrated that NCs can be obtained from cellulose isolated from easily renewable plant feedstocks, such as avocado seeds [1], *Luffa cylindrica* and *Coffea arabica* waste [24], *Miscanthus × giganteus* var. KAMIS [28], *Miscanthus sacchariflorus* (Maxim.) var. Soranovskii [29], palm oil bunches [6,30], pistachio shell [31], bitter bamboo stems [32], tobacco stems [33], oat hulls [34], *Posidonia oceanica* brown algae [35], hemp [36], *Acacia mangium* [37], rhizophora and kenaf fibers [30], Alfa grass [38], and intermediate flax straw [39]. Besides plant-based feedstocks, it has been shown that NCs can be obtained from bacterial (microbial) cellulose that is distinguished by its unique properties and a nanoscale structure [40,41,42]. Most of the cited studies lack complete information on the nitration and stabilization conditions, and comprehensive characterization of NCs.

Despite the extensive diversity and large-scale production of natural cellulose, efforts continue to synthesize it [43] via chemical and enzymatic pathways. The reasons behind the development of this knowledge-intensive field and the history of this process are well described. The growing scientific interest in surpassing nature to synthesize even the most abundant polysaccharide, cellulose, drives the exploration of its functionalization via the most fundamental method—nitration. The present study reports on synthetic cellulose (SC) obtained by electropolymerization [44]. The current literature lacks studies on the use of cellulose of synthetic origin as a precursor of low-esterified NCs, making this work undoubtedly relevant.

The aim of this work was to study the nitration of a new type of cellulose, obtained synthetically via electropolymerization from aqueous glucose solutions, and comprehensively characterize the physicochemical properties, morphology, molecular and supramolecular structure, and thermal stability of both the precursor and its derivative.

## 2. Materials and Methods

### 2.1. Substrate for Study

The synthetic cellulose (SC) [44] provided by OOO “Master Brand” (Moscow, Russia) was used as the substrate for this study. The synthetic method for SC relies on electropolymerization of an aqueous glucose solution in the presence of catalytically active tungsten–vanadium heteropolyacid of the 1–12 series with the chemical formula H_6_[PW_10_V_2_O_40_].

The reagents used were a 20–40 wt.% aqueous glucose solution and a tungsten–vanadium heteropolyacid catalyst at a ratio of 1 L to 1–10 g, respectively. Cellulose is obtained in the form of flakes in a dielectric bath within a temperature range of 25–35 °C by using direct current power supplied to the anode and cathode [44].

#### Physicochemical Analysis of SC

The mass contents of α-cellulose and ash in the SC, as well as the degree of polymerization (DP), were determined by standard methods of cellulose analysis. The mass content of α-cellulose was measured by treating cellulose with a 17.5% NaOH (CAS 1310-73-2, at least 99.0%, Scharlab, Sentmenat, Spain) solution and quantifying the undissolved residue after being washed with a 9.5% NaOH solution and water, followed by drying as per the TAPPI standard [45]. The mass content of ash was measured after cellulose was incinerated in a muffle furnace in accordance with the TAPPI standard [46]. DP was determined by the efflux time of a cellulose solution in cadoxene (cadmium oxide, CAS 1306-19-0, at least 99.0%, OOO Khimreactivsnab, Ufa, Russia, and ethylenediamine, CAS No. 107-15-3, at least 99.0%, manufactured by AO LenReaktiv, Saint-Petersburg, Russia) from a VPJ-3 ECROS viscosimeter (ECROSKHIM Co., Ltd., Moscow, Russia) with a capillary diameter of 0.92 mm, as reported in [47]. The SC wettability was determined and calculated using the formula reported in [48]. A weighed portion of air-dried cellulose was placed in a pre-weighed cylinder with a perforated bottom up to the inner boundary of the cylinder. The cylinder was placed into a container with distilled water at 20 °C, with the water level aligned with the inner boundary of the cylinder. After a 30 s residence time, the cylinder was taken out and weighed. The moisture content of the SC was measured using an Ohaus MB23 moisture analyzer (Pine Brook, NJ, USA).

Prior to nitration, the SC was dried in a Binder ED23 oven (Binder, Tuttlingen, Germany) at 60 ± 5 °C to a residual moisture content of no more than 5%.

The experimental results were obtained in triplicate, statistically processed using standard methods with Microsoft Office Excel 2019, and are reliable.

### 2.2. Nitration and Stabilization of SC

The dried SC weighing 12 g was treated with commercial mixed sulfuric-nitric acids with an initial water content of 14%. Nitration parameters: the mass ratio of SC to mixed acid was 1:50, temperature was 25–30 °C, and the nitration time was 40 min. Nitration was carried out with continuous stirring using an HS-50A-Set overhead stirrer (Witeg Labortechnik GmbH, Wertheim, Germany). The NC obtained from the SC were filtered from the reaction mixture on a Büchner funnel and thoroughly washed with a large amount of distilled water at a temperature of no more than 15 °C. The NC obtained from the SC was then stabilized by sequential boiling in water (1 h), in a 0.03% sodium bicarbonate (CAS 144-55-8, at least 99.0%, OOO Khimleader, Barnaul, Russia) solution (3 h), and again in water (1 h) until the filtrate became neutral when tested against litmus paper.

All stabilization stages were performed at 90–95 °C with continuous stirring. Before analysis, the NC was initially open-air-dried for 24 h at room temperature, then dried in an oven for 1 h at 100 ± 5 °C, weighed to determine the actual yield, and analyzed.

The nitration and stabilization experiments were performed in triplicate and presented as averages.

### 2.3. Calculation of the Actual Yield of SC-Based NC

The actual yield of the NC sample (%) was calculated via Equation (1):

(1) $$W=\frac{m_{NC}}{m_{SC}}\cdot 100$$

where *W* is the actual yield of NC, %; *m_NC_* is the weight of the obtained NC, g; and *m_SC_* is the initial weight of the SC, g.

A similar calculation of the actual yield of NC is reported in [21].

### 2.4. Physicochemical Analysis of SC-Based NC

The nitrogen content was determined using a quantitative method with iron(II) sulfate [23,28,29,39,41,49]. This method is based on the saponification of NC (0.12 g) with concentrated sulfuric acid (CAS 7664-93-9, at least 99.0%, OOO Khimleader, Barnaul, Russia) (25 mL), followed by the reduction of the resulting nitric acid by iron(II) sulfate (CAS 7783-85-9, at least 99.0%, OOO Khimleader, Barnaul, Russia) to nitric oxide (NO). The nitric oxide then reacts with an excess of iron(II) sulfate to form the [Fe(NO)]SO_4_ complex, which imparts a yellowish-pink color to the solution. A weighed sample of NC (0.12 g) was placed in a conical flask (100 mL) and sulfuric acid (25 mL) pre-cooled to 5 °C was carefully added. The flask was sealed with a stopper, placed in a refrigerator, and kept until NC was completely dissolved at about 5 °C. The resulting solution was then titrated with a 0.5 N solution of Mohr’s salt until the yellowish color turned yellowish-pink. The titration was carried out while continuously cooling the solution to a temperature of 3 °C using a bath with a cooling agent. The solubility of NC (1 g) in acetone (50 mL) (CAS 67-64-1, at least 99.0%, OOO Khimleader, Barnaul, Russia) was determined by filtering the NC residue undissolved in acetone, followed by drying and weighing it on an analytical balance [33,50]. The viscosity of NC was determined by measuring the efflux time of a 2% solution of NC (1 g) in acetone (46 mL acetone and 4 mL water) from a VPJ-1 ECROS glass capillary viscometer (ECROSKHIM Co., Ltd., Moscow, Russia) [28,29,39,41,50,51]. The solubility in an alcohol–ether mixture was determined by dissolving NC (1 g) in an alcohol–ether solvent (150 mL, an ethyl alcohol-to-diethyl ether (CAS 64-17-5, at least 99.0%, OOO Khimleader, Barnaul, Russia; CAS 60-29-7, at least 99.0%, OOO Khimleader, Barnaul, Russia, a ratio of 1:2 vol%), followed by filtration, drying, and weighing the undissolved residue on an analytical balance [28,29,39,41,50,51]. The ash content of the NC was determined by the slow decomposition of NC (1 g) with concentrated nitric acid (CAS 7697-37-2, at least 99.0%, Himmedsnab Group, Korolev, Russia) (2–5 mL) upon heating, followed by incineration and weighing of the calcined residue [51]. The reagents used for the NC analysis were supplied by OOO Khimleader (Barnaul, Russia) and OOO Vector (Saint-Petersburg, Russia). The experimental results were obtained in triplicate and processed using standard statistical methods with Microsoft Office Excel 2019 software.

### 2.5. Structural Analysis of SC and SC-Based NC

#### 2.5.1. Scanning Electron Microscopy (SEM) of SC and SC-Based NC

The surface morphology of SC and SC-based NC was evaluated using SEM, which is often used to characterize cellulose and NCs [5,9,23,24,32,35,37,38]. SEM characterization was performed using a JSM-840 (Jeol Ltd., Tokyo, Japan) after spraying a 1–5 nm thick Ag layer. The study was conducted at magnifications of ×100 to ×10,000.

#### 2.5.2. IR Fourier Spectroscopy of SC and SC-Based NC

The molecular structures of SC and SC-based NC were studied using IR Fourier spectroscopy [1,9,23,24,32,35,37,38]. IR spectra were recorded using an InfraLum FT-801 FT-IR spectrophotometer (OOO NPF Lumex Sibir, Novosibirsk, Russia) in the frequency range of 4000–500 cm^−1^. To record the spectra, tablets were pressed in potassium bromide at a ratio of SC/NC:KBr = 1:150. The spectra were normalized to the absorption band at 1162 cm^−1^, corresponding to the C-O-C glycosidic linkage stretching vibration in cellulose [24].

#### 2.5.3. X-Ray Diffraction Analysis (XRD) of SC and SC-Based NC

The X-ray diffraction patterns of the studied SC and SC-derived NC were obtained on a DRON-3M diffractometer (The Bourevestnik, JSC, Saint-Petersburg, Russia) in reflection and transmission geometries with FeKα radiation, monochromatized by a pyrolytic graphite crystal placed in the primary beams. The X-ray diffraction profiles were scanned over a 2θ range from 3° to 90°, with a step size of 0.1°. The signal counting time per point was 10 s. Since the NC sample was isotropic, the diffraction patterns recorded in reflection and transmission geometries were identical; therefore, only the reflection-mode pattern is presented in this article. A full-profile analysis to determine the content of the Iα or Iβ allomorphs in the NC was performed following the reported guidelines [52,53,54,55,56,57] and methodologies [57,58,59]. The lattice parameters and the atomic lattice model of the NC were determined using the data for the two known models: cellulose Iα [52,57] and cellulose Iβ [53,54,57]. Based on the obtained diffractograms, the crystallinity index (CrI) and the crystallite sizes were calculated. The CrI was determined using a modified Ruland method, which is based on separating the peaks corresponding to scattering from the crystalline component of biopolymers and the maximum of diffuse scattering from the amorphous component, followed by fitting the profiles of the calculated and experimental curves [54,55,56,57,58,59]. The size of the crystalline regions (mosaic blocks, coherent scattering domains (CSD)) was calculated using the Scherrer equation [54,57]:

(2) $$\beta_{Scherrer}=\frac{0.94\lambda }{D_{hkl}\cdot cos\theta }$$

where *β*_Scherrer_ is the line broadening due to the CSD sizes; *λ* is the X-ray wavelength; *D_hkl_* is the sought-for effective size of the CSD in the direction normal to the reflecting planes with indices *hkl*; and *θ* is the Bragg angle. Under the Gaussian and Cauchy approximations, the *β*_Scherrer_ values were calculated using the formulas:

$${\beta_{Scherrer}^{2}=B^{2}-b^{2}\;and\;\beta }_{Scherrer}=B-b,\;\mathrm{respectively}$$

where *B* and *b* are the full widths at half maximum (FWHM) of the diffraction peaks for the sample and the standard, respectively. The CSD sizes calculated using these two approximations define the range within which the true values lie. For further analysis, the average values were used.

#### 2.5.4. Coupled Thermogravimetric and Differential Thermal Analysis (TGA-DTA)

The thermal behavior of SC and SC-based NCs [4,9,28,29,32,35,38,39] was investigated using a TGA/DTG-60 thermogravimetric analyzer. The weight of each sample was 0.5 mg. The experiment was conducted under a nitrogen atmosphere in a temperature range from 0 °C to 600 °C (with a maximal temperature of 350 °C) at a heating rate of 10 °C/min.

## 3. Results and Discussion

### 3.1. Physicochemical Properties of the SC Sample

**Figure 1:**
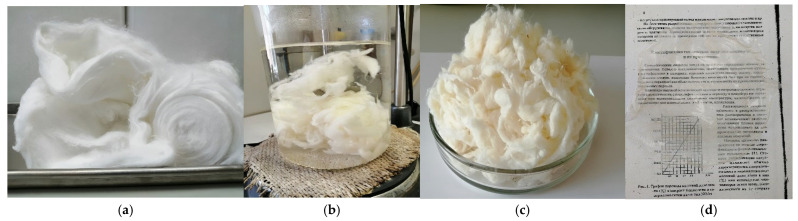
Photographs of (**a**) the initial SC, (**b**) the high-temperature treatment of the obtained NC, (**c**) the target product, SC-derived NC, and (**d**) a transparent film obtained after the NC solution in an alcohol–ether solvent was air-dried.

Visually, the SC appears as a loose, long-fibrous mass of white color, free of foreign inclusions and other non-cellulosic impurities (Figure 1a). In appearance, the SC is virtually indistinguishable from cotton cellulose.

Table 1 presents the properties of the SC in comparison with cotton and wood celluloses [22,25], which are typically used for producing the highest-quality NCs with a wide range of characteristics for various applications.

It follows from the data presented in Table 1 that the SC is of very high quality. Its α-cellulose content reaches 99.4%, a value comparable only to that of the highest-grade CC (99.0%) and exceeding that of the first-grade CC (97.2–98.0%), CC (98.0%) from [25], and wood cellulose of both grades (WC S 93.0% and WC RP 94.0%). The ash content of the SC is 0.24%, which is comparable to that of the first-grade CC and WC RP (0.20%). Table 1 presents the range of possible DP (degree of polymerization) values for commercial grades of cotton and wood celluloses. The DP of 3140 for SC is quite high and exceeds that of specific grades of cotton cellulose (e.g., 1000, 1300, 1900, and 2100) and wood cellulose (e.g., 460). However, SC is about 2.2–2.5 times inferior in wettability to cotton and wood celluloses, at 59 g versus 135–150 g, which may be attributed to the denser fiber packing of SC. 

It is noteworthy that the SC is superior in α-cellulose content to celluloses derived from other plant-based sources, such as: avocado seeds (89.69%) [1], *Luffa cylindrica* (90.29%) and *Coffea arabica* waste (80.70%) [24], *Miscanthus × giganteus* var. KAMIS (95.60%) [28], *Miscanthus sacchariflorus* (Maxim.) var. Soranovskii (94.54%) [29], oil palm bunches (98.9%) [30], bitter bamboo stems (94.00%) [32], *Acacia mangium* pulp (>80%) [37], rhizophora (91.3%) and kenaf fibers (91.60%) [30], and intermediate flax straw (87.80%) [39].

Given the stringent requirements for dissolving cellulose—specifically, an α-cellulose content of no less than 95% [21]—the achieved high quality of the obtained SC suggests its strong potential for successful chemical modification into NC with satisfactory functional properties.

### 3.2. Physicochemical Properties of SC-Based NC

Figure 1 displays photographs of (a) the initial SC, (b) the high-temperature treatment of the obtained NC, (c) the target product, SC-derived NC, and (d) a transparent film obtained after the NC solution in an alcohol–ether solvent was air-dried. In appearance, the NC (Figure 1c) differs from the SC in color and shape. Unlike the initial SC, the NC has a creamy hue, and its fibers are intertwined into denser formations.

Table 2 presents the actual yield of the SC-derived NC and its main functional properties in comparison with the literature data for NCs from cotton cellulose according to the Chinese and Russian classifications [21], as well as with data for NCs from cotton cellulose [32], commercial microcrystalline cellulose (MCC) [50], and MCC from avocado seeds [1], since there is no information on high-viscosity NCs from other feedstocks that could be used for comparison.

The actual yield of the synthesized NC was calculated based on the weight of the initial air-dried SC and amounted to 138% [21]. This is due to an increase in the average molecular weight of the cellulose monomer unit due to the substitution of the hydrogen atom of the hydroxyl group (-H-OH) in cellulose by the nitro group (-NO_2_) [21]. The obtained data align with the classical concept of the actual yield of NC, with a 1.5–1.7-fold increase in the target ester mass, and indicate SC stability in the nitrating mixture.

The obtained NC is characterized by the following key functional properties: a nitrogen content of 11.83%, a viscosity of 198 mPa·s, a solubility of 90% in an alcohol–ether solvent, and an ash content of 0.05%. The NC synthesized from the SC exhibited 100% solubility in acetone, confirming its classification as a nitrate ester of cellulose [21]. After solubility measurement, the NC alcohol–ether solution was dried out in a thin layer on a flat surface. The dry film had a homogeneous texture, a smooth and sufficiently even surface, and high transparency (Figure 1d), corroborating the high solubility of the NC. Notably, a text in 12-point font is easily readable through the film.

A comparison of the synthesized NC with the literature data on NCs from cotton cellulose (CC) [21] (Table 2) indicates that they are comparable in terms of the nitrogen content (11.83% vs. 11.56–12.19%), with only a minor difference in the solubility in an alcohol–ether solvent, 91% vs. 98–99.2%. Furthermore, the SC-based NC is significantly superior in the ash content to CC-based NCs (0.05% vs. 0.2–0.5%), as the ash content of the NC adversely affects the quality of films [21]. The comparison of the SC-based NC with the literature data for CC-based NCs [32] (Table 2) revealed that the SC-based NC is inferior in the nitrogen content to CC-based NCs (11.83% vs. 12.50%) but better in terms of the ash content (0.05% vs. 0.19%). The reason for the elevated viscosity value (198 mPa·s) of the obtained synthetic cellulose nitrates, compared to the literature data (Table 2), is the high degree of polymerization (3140) of the initial synthetic cellulose, as well as the absence of a high-temperature autoclaving treatment of these SC-derived NCs. Compared to CC-based NCs, the high viscosity of the SC-based NC significantly widens the potential application range, from rocket propellants to biomedicine. The high viscosity of the SC-based NC suggests that lower concentrations of the solution can be used to achieve the desired fluidity—for instance, in the film-forming processes. Furthermore, the high viscosity indicates its high thermal stability—which is directly dependent on viscosity. Moreover, if needed, the use of favorable autoclaving conditions for SC-based NCs [34] can guarantee not only a decrease in the viscosity to the recommended values, but also an increase in the solubility in alcohol–ether solvents. It is important to note that despite the low wettability value of the SC (59 g) compared to CC (135–150 g) (Table 1), the nitration of the SC was quite successful, as confirmed by the satisfactory properties of the functionalized NC. It might be that such a low wettability of the SC compared to CC is attributed to the specifics of the SC synthesis method.

The comparison of the key functional properties of the SC-derived NC with those of commercial MCC-based NC [50] (Table 2) reveals that the SC-based NC exhibits a comparable solubility in the alcohol–ether mixture (91% vs. 90%), despite a clear superiority in viscosity (198 mPa·s vs. 17 mPa·s), which is highly advantageous for potential applications of the SC-based NCs—for example, as a component of rocket propellants and composites, as well as in dental materials, analytical medicine, and in the manufacture of anti-COVID masks and membrane filters for bacterial removal [4,5,6,7,8,11,12,13,14,15,16,17,18,19,20]. The substantial difference in viscosity is attributed to the significantly higher degree of polymerization of the SC (DP 3140) compared to that of commercial MCC (DP 350) [60]. A similar trend of comparable solubility in the alcohol–ether solvent (91% and 90–93%) is observed when comparing the SC-based NC with the NC from avocado seed-derived MCC [1] (Table 2), with a minor difference in the nitrogen content (11.83% vs. 12.23–12.26%).

### 3.3. SEM Results for SC and SC-Based NC

**Figure 2:**
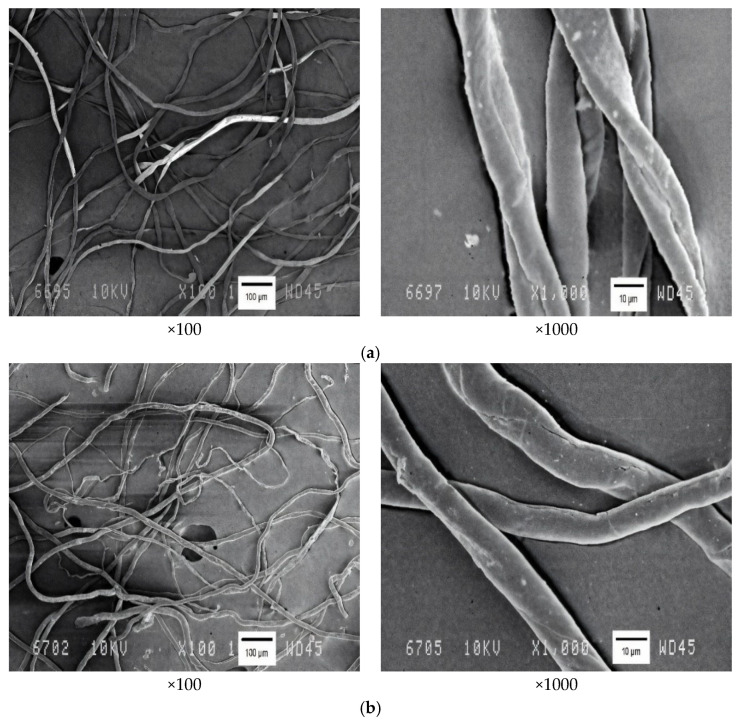
SEM images of (**a**) SC and (**b**) SC-based NC.

Figure 2 shows SEM images of the SC and SC-based NC.

The SEM micrographs of the SC (Figure 2a) show flat, curly fibers with a smooth surface of approximately 10–20 μm in diameter, with no fiber aggregation. The morphology and diameter of the SC fibers are very similar to those of cotton cellulose (CC) [23,24,32], with the exception that tubular fibers characteristic of natural CC are absent. The absence of the aggregated SC microfibrils may be attributed to the high quality of the SC, as corroborated by the high values of the α-cellulose content (99.4%) and the degree of polymerization (DP 3140). As a result of nitration, the fibers of the SC-derived NCs acquire a cylindrical shape with a diameter of approximately 25 μm (Figure 2b). Furthermore, their surface exhibits the formation of the microcracks and roughness that result from the swelling and unravelling of the original cellulose fibers when esterified. The morphology of the synthesized NC is similar to that of commercial NCs, with an average fiber diameter of approximately 20 µm [2,32,38], and is distinct in exceptional homogeneity from NCs derived from alternative plant feedstocks [1,28,32,35,38].

The first-ever results on the morphology of cellulose of synthetic origin broadly confirm the fundamental alterations of classical cotton cellulose. This, in turn, suggests the promising potential for producing nitrates from synthetic cellulose.

### 3.4. FT-IR Spectroscopy Results for SC and SC-Based NC

**Figure 3:**
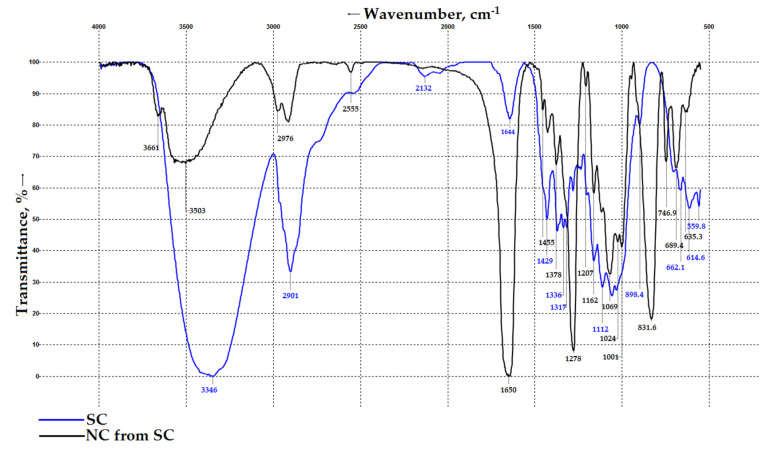
FT-IR spectra of the SC and SC-based NC samples.

Figure 3 depicts FT-IR spectra of the SC and SC-based NC samples. The spectra were normalized to the absorption band at 1162 cm^−1^, corresponding to the C-O-C glycosidic linkage stretching vibration in cellulose. This band was selected for normalization because this functional group remains unaffected and is not involved in the substitution reaction [24].

The FT-IR spectrum of the initial SC (Figure 3) exhibits the main absorption bands at 3346, 2901, 1644, 1429, 1162, and 1112 cm^−1^. These bands are assigned to O-H stretching, C-H asymmetric and symmetric stretching, O-H bending of absorbed water, CH_2_ asymmetric bending, C-O-C stretching, C-O skeletal stretching, and the β-glycosidic linkage vibration, respectively [61]. As expected, no absorption bands at 1735 cm^−1^ (a stretching vibration of the carbonyl group of hemicelluloses) or 1512 cm^−1^ (aromatic ring vibration of lignin) were detected in the SC spectrum, confirming the absence of non-cellulosics, such as hemicelluloses and lignin [62,63,64]. Thus, the FT-IR spectrum confirms the purity of the SC and its similarity to commercial cotton cellulose (CC) [23,24,32]. Furthermore, the SC spectrum shows a clear resemblance to the spectra of celluloses derived from other plant-based sources [24,28,32,35,38].

Following SC functionalization, the NC spectrum (Figure 3) shows a decrease in the intensity of the bands corresponding to the O-H stretching (3503 cm^−1^) and C-H asymmetric/symmetric stretching (2976 cm^−1^), indicating the formation of new chemical groups [1]. New absorption bands appear: two intense bands at approximately 1650 cm^−1^ and 1278 cm^−1^, which are assigned to the asymmetric and symmetric stretching of the NO_2_ group, and a broader, intense band around 832 cm^−1^ attributed to the NO_2_ stretching, as well as two less intense bands at 747 cm^−1^ and 689 cm^−1^, corresponding to the asymmetric and symmetric bending vibrations of O-NO_2_ [24]. The obtained spectral data are in good agreement with the FT-IR spectroscopy data reported for commercial CC-derived NCs [24,35,38], commercial NCs [1,2,32], and NCs from alternative plant feedstocks [1,24,28,29,32,35,38,39]. It should be noted that most studies report the identification of NC from all five peaks [1,4,24,28,35,38], whereas others note the presence of only three peaks near 1650, 1275, and 825 cm^−1^ [15,32]. Thus, this study demonstrates no principal differences between natural celluloses and the cellulose synthesized via electropolymerization, confirming the positive forecasts made in the review [43] regarding the chemical synthesis of cellulose. Further, it should be noted that the commonly used nitration method guarantees the synthesis of high-quality NCs from the SC, just like from natural celluloses. Moreover, the FT-IR spectrum of the SC-derived NC shows no absorption bands at 2300 cm^−1^ corresponding to NC decomposition products.

### 3.5. XRD Results for SC and SC-Based NC

The supramolecular structure is one of the main factors governing the properties of cellulose and its modification products [54,55,56,57,65,66]. Cellulose I, the most prevalent form in nature, is a composite of two distinct crystalline allomorphs: Iα and Iβ. The Iβ allomorph is dominant in higher plants, including cotton cellulose [54,57]. According to the review [43], the chemical synthesis of cellulose that fulfills three key conditions—linkage regioselectivity (only 1→4 linkages and the absence of di-substitution that would lead to branching), stereoselectivity (the anomeric carbon having a strict β-configuration), and the DP control (precise regulation of chain length or low dispersity)—would yield cellulose consisting solely of the Iβ allomorph. A comparison of the diffractogram of the cellulose under study with the data on the Iα and Iβ allomorph models allows for the preliminary identification of the type of allomorph [55,56,57]. Consequently, new insights into SC nitrates acquire particular importance, as they can either confirm or challenge the established views on principal alterations in the polymer’s crystalline structure upon functionalization of the surface with bulky substituents [61].

For these reasons, the supramolecular structure of the SC and the SC-derived NC was investigated using X-ray diffraction (XRD), and a quantitative analysis of the crystalline structure parameters of both materials was performed.

**Figure 4:**
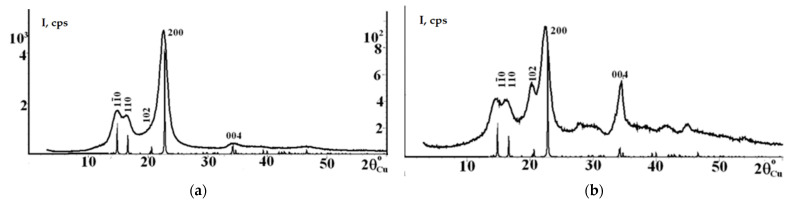
X-ray diffractograms recorded in (**a**) reflection and (**b**) transmission geometries. Indices of reflections for the Iβ allomorph are indicated.

Figure 4 depicts the X-ray diffractograms of the SC recorded in both reflection and transmission geometries.

The X-ray diffractograms of the SC (Figure 4a,b) exhibit four main diffraction peaks at 2θ values of about 14.8°, 16.3°, 22.3°, and 34.6°. These peaks are assigned to the [$1\overset{–}{10}$], [110], [200] and [004] crystallographic planes characteristic of the cellulose Iβ allomorph [55,59,66]. Both diffractograms show a comparison of the experimental XRD pattern of the SC sample under study with the theoretical one for cellulose Iβ. That is, the most intense diffraction peaks in the experimental curve coincide with the peak positions in the theoretical pattern for the cellulose Iβ model, indicating that the structure of the sample under study corresponds to the cellulose Iβ allomorph. Similarly, studies on cotton linter [54] and cotton cellulose (CC) [66] have reported analogous findings, concluding a predominant cellulose Iβ crystalline structure in both samples based on the alignment of peaks in their reflection-mode diffractograms. Consequently, a comparison of our SC data with the results from [54,66] reveals a clear coincidence of the intense reflections. This confirms that the experimental SC sample corresponds not only to the cellulose Iβ model but also aligns with the published data for cotton linter and CC.

The coincidence of the diffraction peaks and the conclusions regarding the monoclinic Iβ unit cell in cellulose samples from banana rachis and commercial cellulose are discussed in [67]. The same study also describes the triclinic Iα unit cell in bacterial cellulose for comparison. Despite differences in experimental details and equipment used, all these results were obtained using XRD, similarly to methodologies reported in [58,59]. It should be noted that since it is well-established that cotton cellulose (CC) consists chiefly of the cellulose Iβ allomorph or the monoclinic phase of cellulose [57,59], a full-profile analysis of the structure of CC samples is rarely reported. Furthermore, since it is well-known that many celluloses isolated from plant-based sources [24,57,67] are the cellulose Iβ allomorph, the XRD results reported in those studies generally confirm the factual existence of merely the cellulose I allomorph, with the Iβ structure being the default assumption.

Table 3 summarizes the results of the structural refinement of the SC for the Iβ allomorph, showing the crystallographic characteristics and reliability factors averaged over all recorded curves. Refinement using the data on the Iα allomorph did not yield a satisfactory fit.

Due to the lack of published unit cell parameter data for synthetically derived cellulose samples, the results outlined in Table 3 can only be compared with analogous data for natural celluloses [59]. As is known, there are three models of cellulose Iβ, which differ from each other in the relative orientation of adjacent molecules [59]: antiparallel, parallel-up, and parallel-down. In our case, a full-profile analysis showed that the SC sample has a monoclinic unit cell with an antiparallel chain arrangement and the following parameters: *a* = 7.952(1) Å, *b* = 8.167(4) Å, *c* = 10.35(2) Å, and a monoclinic angle *γ* = 96.3(1)°. The reliability factors were R_wp_= 7.5% and R_p_ = 5.4%. These values are consistent with the crystallographic characteristics of the Iβ unit cell (*a* = 7.784 Å, *b* = 8.201 Å, *c* = 10.38 Å, *γ* = 96.55°) reported in [52,57], as well as with data on the bleached hardwood sulfate pulp, especially considering that the XRD approaches and methodologies used are similar [59]. Compared to the crystallographic characteristics of bacterial cellulose representing the Iα allomorph [58], the unit cell parameters of the SC under study (Table 3, reflection geometry) for the parameters *a*, *b* and *γ* are significantly larger (6.78 Å, 5.92 Å and 80.4°, respectively), while the parameter *c* almost coincides with 10.49 Å.

Table 4 presents the crystallinity index (CrI) and crystallite size (*D_hkl_*) calculated from the diffractograms recorded in reflection and transmission geometries.

The calculated crystallinity index (CrI) of the SC showed that the values obtained from the reflection- and transmission-mode diffractograms are close to each other and are 81% and 86%, respectively. Due to the lack of XRD data on synthetic celluloses, our findings may be compared with published data for natural cellulose samples. For example, cotton linter with DP 4300 has a CrI of 58% [54], classical cotton cellulose (CC) with DP 2140 has a CrI of 86.68% [65], and a CC sample has a CrI of 75.52% [24]. The CrI values of cellulose isolated from various plant feedstocks chiefly exceed 50%: for example, 75.05% for *Luffa cylindrica*, 59.65% for *Coffea arabica* waste [24], 61.2% for bitter bamboo stems [32], 60.5% for *Posidonia oceanica* brown algae [35], 73% for Alfa grass [38], and 72% for hardwood [59]. Furthermore, the CrI of bacterial cellulose is known to vary widely, between 55% and 100%, and is generally dependent on the cultivation conditions [40,58]. Thus, the CrI value obtained for the SC by the widely used Segal method [56] indicates a high proportion of the crystalline component [57] in the synthesized cellulose compared to natural celluloses, suggesting that the electropolymerization of cellulose from an aqueous glucose solution using a catalytic tungsten–vanadium heteropolyacid of the 1–12 series yields highly crystalline cellulose.

In light of the above, the crystallite sizes of the SC are of particular interest. As shown in Table 4, the crystallite sizes (*D_hkl_*) are independent of the measurement geometry (reflection or transmission) and are 43–44 Å along the [$1\overset{–}{10}$] direction, 62–63 Å along [110], 58–59 Å along [200], and 94 Å along [004]. The obtained data are close to the values reported for cotton linter with DP 4300 (49 Å [$1\overset{–}{10}$], 65 Å [110], 58 Å [200]) [54]. This confirms the similarity between the experimental SC and natural CC, which is also evidenced by the predominant Iβ allomorph, the unit cell parameters, and the high CrI value. The crystallite sizes (*D_hkl_*) of the SC differ from those reported for cellulose samples isolated from various alternative plant feedstocks. For instance, the crystallite size of cellulose from bitter bamboo stems in the [200] direction is smaller than that of the SC and is 32 Å [32], while the crystallite sizes (*D_hkl_*) of cellulose from Alfa grass [68] are coincident in the three main directions (40 Å [$1\overset{–}{10}$], 40 Å [110], 40 Å [200]) but are smaller than those of the SC.

Thus, for the first time, a full-profile analysis has discovered that the SC represents a monoclinic cellulose Iβ phase with an antiparallel chain arrangement. The CrI ranges from 81% to 86%, with unit cell parameters *a* = 7.952 Å, *b* = 8.167 Å, *c* = 10.35 Å, a monoclinic angle of 96.3°, and crystallite sizes of 44 × 62 × 59 × 94 Å. The obtained XRD data for the SC are unique in the published literature and show a similarity to those reported for high-molecular-weight cotton cellulose (CC).

**Figure 5:**
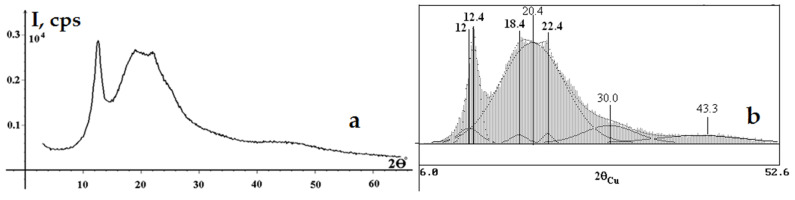
(**a**) X-ray diffractogram of SC-derived NC in reflection geometry and (**b**) deconvolution of the diffraction pattern showing the peak positions for the crystalline (black font) and amorphous (regular font) components.

Figure 5 shows (a) the X-ray diffractogram of the NC sample derived from the SC, recorded in reflection geometry and (b) the deconvolution of the diffraction pattern of the SC-based NC.

The X-ray diffractogram of the SC-derived NC (Figure 5a) exhibits a typical X-ray profile of NC [1,2,4,24,32,35,38,61], featuring new diffraction peaks at 2θ = 12.4° and 20.4° relative to the SC. These peaks are associated with ordered domains and amorphous regions of the NC chains, respectively [1,35,38,61]. The results of the diffraction pattern deconvolution and the peak positions of the crystalline and amorphous components (Figure 5b) show a broad amorphous halo centered at 2θ = 20.4° and patterns for each crystalline peak, including those at 12.0°, 12.4°, 18.4°, and 22.4° that disappear after nitration of crystalline cellulose [35,66]. Regardless of the deconvolution method used, the diffraction patterns of the functionalized cellulose samples are fundamentally distinct from those of the precursors and coincide with each other [35,38,56,66]. The diffractogram profiles, the number of peaks and their shapes (2θ) obtained in the present study show good agreement with the XRD data and deconvolution results reported for the CC-derived NC [38], providing further evidence of the similarity between the SC and CC.

The significant broadening of the peak corresponding to the [200] crystallographic plane and the disappearance of the [$1\overset{–}{10}$], [110], and [004] planes are mandatory explicit changes in the crystalline structure of the NC compared to the initial SC (Figure 4a). Furthermore, the diffractogram profiles show little difference from each other, independent of the nitrogen content in the NC [2,24]. Therefore, the diffractogram of the SC-derived NC is similar to both NCs from cotton cellulose (CC) [2,24,32,35,38] and NCs from alternative sources, such as avocado seeds [1], *Luffa cylindrica* and *Coffea arabica* [24], bitter bamboo stems [32], *Posidonia oceanica* brown algae [35], and Alfa grass [38]. An analysis of the published literature on commercial CCs reveals scarce examples of comparing the diffractograms of the initial cellulose and NCs [24,32,61]. However, the study [24] reported interesting results, discussing the hypothesis of a change in the crystallographic plane spacing (*d_hkl_*) upon nitration of cellulose. Using three cellulose samples, such as cotton cellulose (CC), *Luffa cylindrica* and *Coffea arabica* celluloses, it was demonstrated that the crystallographic plane spacing in the [101] direction increases for NCs during nitration compared to the initial cellulose: from 5.87 Å to 6.99 Å for CC and from 6.03 Å to 7.12 Å for *Luffa cylindrica*, while the *d_hkl_* remained almost unchanged for *Coffea arabica* (5.64 Å before and 5.65 Å after nitration).

Table 5 presents the crystallinity index (CrI) and crystallite size (*D_hkl_*) for the SC-derived NC, calculated from the diffractogram in reflection (Figure 5b).

The broadening of the diffraction peaks on the diffractogram of the SC-derived NC (Figure 5b) indicates a decrease in the CrI and evidences amorphization of the NC structure compared to the diffractogram of the initial SC (Figure 4a) [35,38,61]. Based on the reflection-mode diffractogram, the CrI of the SC-based NC was calculated to be 17% (Table 5). Thus, the CrI of the initial SC was found to decrease from 81–86% to 17% upon nitration. The obtained results align with the classical understanding of the change in the crystalline structure of cellulose upon nitration [2,4]. The high-molecular-weight fibers are tightly packed in the SC, providing a higher CrI. The SC fiber molecules undergo heterogeneous rearrangement during nitration, accompanied by a reduction in packing density and the disruption of strong hydrogen bonds within the cellulose structure, a phenomenon similar to that described for CC in [40]. The cleavage and fragmentation of the crystalline domains are accompanied by the substitution of the hydroxyl groups by nitrate ester groups (O-NO_2_). It is these changes that considerably weaken the strong inter- and intramolecular hydrogen bonds responsible for the crystalline state of cellulose chains [35,59,69,70]. Analysis of the published data shows a general trend of decreasing CrI in the initial cellulose, regardless of the cellulose source and XRD methodology features. For instance, it was demonstrated that the nitration of cotton cellulose (CC) reduces the CrI from 68.5% to 24.63% [61]; the nitration of cellulose from bitter bamboo stems decreases the CrI from 61.2% to 12.3% [24]; the nitration of cellulose from *Posidonia oceanica* brown algae reduces the CrI from 60.5% to 23.1% [35], and the nitration of Alfa grass cellulose decreases the CrI from 73.00% to 28.90% [38].

As can be seen in Table 5, the crystallite sizes of the initial SC (Table 4) decrease from 44 × 62 × 59 × 94 Å to 29 × 62 × 26 × 38 Å upon nitration. Analysis of the published data revealed that, despite the multitude of studies on the synthesis of NCs from both classical cotton cellulose (CC) and cellulose from alternative sources, there is no information available on the crystallite sizes (*D_hkl_*) of NCs.

Thus, for the first time, the XRD analysis has provided new insights into the nitration of synthetic, rather than natural, cellulose. This study discovered that the SC undergoes amorphization, accompanied by a decrease in the CrI from 81–86% to 17% and by a reduction in crystallite sizes from 44 × 62 × 59 × 94 Å to 29 × 62 × 26 × 38 Å. No similar results can be found in the published literature.

### 3.6. TGA/DTA Results for SC and SC-Based NC

**Figure 6:**
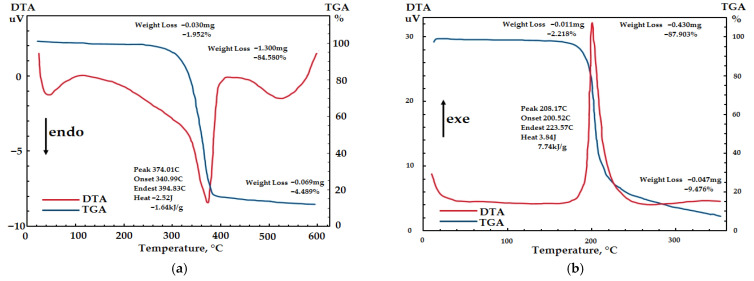
TGA curves of (**a**) SC and (**b**) SC-derived NC.

Figure 6 shows the results of coupled TGA/DTA analyses of SC and SC-based NC.

The experimental TGA/DTA curve of the SC (Figure 6a) shows three characteristic TGA regions. The first region, from the onset temperature of the experiment to 120 °C, is where the SC is dried out with a minor weight loss of 2% due to the evaporation of moisture and other volatiles contained in the sample [61,69]. The second region is between 120 °C and 410 °C, where the cellulose crystals undergo pyrolysis with a weight loss of 84%. This region corresponds to depolymerization, dehydration, and the cleavage of 1,4-glycosidic bonds, followed by the formation of a charred residue [61]. The third region, within the temperature range of 410 to 500 °C, is where the cellulose continues to decompose with a minor weight loss of 4%. The onset temperature of intense decomposition was 341 °C. The endothermic peak at 374 °C in the DTA curve of the SC (Figure 6b) is accompanied by a heat release of 1.64 kJ/g. The obtained thermogram corresponds to a typical decomposition curve of commercial CC, with an endothermic peak around 360–370 °C [71], and confirms the similarity to CC, which has excellent thermal stability and purity. It should be noted that the high thermal stability of the SC ensues from its high-polymer nature (DP 3140).

According to the TGA thermogram (Figure 6b), the main thermal degradation of the SC-derived NC begins at about 200 °C and continues up to 260 °C, with an NC weight loss of 88%. Further, the NC continues to decompose with a minor additional weight loss, leaving a final residue of 9%. The DTA curve (Figure 6b) exhibits a single narrow exothermic peak with a temperature of about 208 °C. This corresponds to the thermolytic cleavage and release of the O-NO_2_ group [35,38,70], triggering an autocatalytic reaction and accelerating the decomposition of the NC [61]. The obtained data align with the thermal characteristics of commercial NCs [4,9,35,61]. A comparison of the onset temperatures of intense decomposition between the synthesized SC-based NC and commercial NCs shows a proximity of their values (200 °C vs. 181 °C [4], 198 °C [9], and 195 °C [35]), indicating that they are similar in thermal stability, high chemical purity, and energy content. The high thermal stability ensues from the high viscosity of the SC-based NC [72]. The SC-derived NC exhibits a high specific heat of decomposition of 7.74 kJ/g, confirming the high-energy nature of the polymer [73]. This property can be exploited in relevant economic sectors. The obtained data are also in good agreement with the TGA/DTA results reported for NCs derived from alternative feedstocks [28,29,32,35,38,39], which in turn validates the quality of the NC obtained from synthetic rather than natural cellulose [43].

### 3.7. Potential Applications of Synthetic Cellulose Nitrates

All experimental data are summarized in Table 6.

It is well known that directly comparing research findings from different scientific schools is difficult due to differences in methods and equipment used. However, Table 6 allows the comparison between the results of this study and those of other studies, as well as reference natural celluloses. 

The synthesized synthetic cellulose nitrates, owing to their high viscosity, can be recommended for use in various sectors of the economy—as a component of formulations used in the mining industry, in novel composites, in controlled energetic materials, in dentistry and cosmetology, in analytical medicine, in immunoassays, as well as in the manufacture of anti-COVID masks and membrane filters designed to detect bacteria trapped in air conditioners, and for drinking water purification.

## 4. Conclusions

This work presents the first comprehensive study of synthetic cellulose (SC) with a long-fibrous structure that features a high α-cellulose content of 99.4% and a high degree of polymerization (DP) of 3140. According to the full-profile analysis, the SC refers to the monoclinic phase of cellulose Iβ with an antiparallel chain arrangement. The unit cell parameters were determined to be *a* = 7.952 Å, *b* = 8.167 Å and *c* = 10.35 Å, with a monoclinic angle of 96.3°, a crystallinity index (CrI) of 81–86%, and crystallite sizes of 44 × 62 × 59 × 94 Å. While the structure and properties of the SC, including thermal stability, are not described in the literature, our results demonstrate the similarity to high-molecular-weight cotton cellulose (CC).

Nitration of the SC allowed the synthesis of NC in an actual yield of 138%, with a nitrogen content of 11.83%, a viscosity of 198 mPa·s, a high solubility of 91% in an alcohol–ether solvent, and a low ash content of 0.05%, evidencing the exceptional quality of the SC-derived NC.

The precursor—the SC—and the SC-based NC were comparatively investigated herein by SEM, FT-IR spectroscopy, XRD, and coupled TGA/DTA. The morphological structure of the SC consists of flat, curly fibers with a smooth surface approximately 10–20 µm wide, exhibiting no aggregation. After nitration, the SC-based NC fibers acquire a cylindrical shape with an enhanced diameter of up to 25 µm, and their surface is covered with numerous microcracks and roughness.

FT-IR spectroscopy of the SC revealed the presence of the main functional groups characteristic of classical cellulose (3346, 2901, 1644, 1429, 1162, and 1112 cm^−1^), and the spectrum of the SC-based NC revealed five new peaks at 1650, 1278, 832, 747 and 689 cm^−1^, indicating that the synthesized polymer refers to nitrate esters of cellulose.

It was found that the SC undergoes amorphization upon nitration, with the CrI falling to 17% and the crystallite sizes decreasing from 44 × 62 × 59 × 94 Å to 29 × 62 × 26 × 38 Å, which is consistent with the fundamental understanding of classical cellulose functionalization. Coupled TGA/DTA revealed a high-temperature endothermic peak of decomposition at 374 °C and a weight loss of 84%, confirming the excellent thermal stability and purity of the SC, thanks to the high DP. The SC-derived NC exhibits a high onset temperature of intense decomposition at 200 °C and a maximal exothermic peak at 208 °C, indicative of high thermal stability. The weight loss of 88% and the high specific heat of decomposition of 7.74 kJ/g corroborate a high chemical purity and the energetic nature of the SC-derived NC.

This study has yielded new knowledge; more specifically, the structure and properties of synthetic cellulose (SC) before and after nitration were determined, with the findings discussed and compared with both classical cotton cellulose and cellulose isolated from alternative plant feedstocks. The experimental results are in full agreement with the fundamental concepts of the functionalization of high-polymer cellulose, not of natural origin, but produced via a synthetic route.

Thus, it has been established that SC and SC-derived NCs fit well within the scientific framework of the chemistry of cellulosic materials.

## Figures and Tables

**Table 1:** Properties of SC compared to cotton and wood celluloses.

Sample Name	* Mass Content, %		DP	Wettability, g	Ref.
	α-Cellulose	Ash			
SC	99.4 ± 0.4	0.24 ± 0.05	3140 ± 10	59 ± 5	present study
CC (highest-grade)	(98.2–99.0) ± 0.4	0.10 ± 0.05	(1000–5000) ± 10	150 ± 10	[22]
CC (first grade)	(97.2–98.0) ± 0.4	0.20 ± 0.05	(1000–5000) ± 10	140 ± 10	[22]
CC	98.0 ± 0.4	no data	2700 ± 10	no data	[25]
WC S	93.0 ± 0.4	0.16 ± 0.05	(400–5500) ± 10	135 ± 10	[22]
WC RP	94.0 ± 0.4	0.20 ± 0.05	(400–5500) ± 10	no data	[22]

Note: * on an oven-dry basis; CC—cotton cellulose; WC S—wood cellulose in the form of strands and WC RP in the form of rolled paper; DP—degree of polymerization.

**Table 2:** The actual yield of SC-based NC and its main functional properties as compared to the literature data for NCs from cotton cellulose (CC) and microcrystalline cellulose (MCC).

Sample Name	Actual Yield, %	Nitrogen Content, %	Viscosity of the 2% Solution in Acetone mPa·s (°E)	Solubility in Alcohol–Ether Solvent, %	Ash Content, %	Ref.
NC from SC	138 ± 2	11.83 ± 0.05	198 ± 2 (32.4)	91 ± 2	0.05 ± 0.01	present study
“H”-type NC	~142	11.75–12.09	8.5–12.3 (1.9–2.6)	at least 98	at most 0.5	[21]
Film NC	~142	11.38–12.00	5–20 *	at least 99.2	at most 0.3	[21]
NC for spray paint	~142	11.56–12.19	0.25–40 *	at least 98.5	at most 0.2	[21]
Commercial NC	no data	12.50	42 ± 2	no data	0.19	[32]
NC from commercial MCC	no data	12.50	17 (3.2)	90	no data	[50]
NC from avocado seed-derived MCC	no data	12.23	no data	90.36 ± 1.91	12.76 ± 1.99	[1]
	no data	12.26	no data	93.28 ± 2.34	9.80 ± 2.20	

Note: * viscosity is given in seconds.

**Table 3:** Crystallographic characteristics of SC (unit cell parameters) and reliability factors (R_wp_, R_p_).

Sample	Geometry	*a*, Å	*b*, Å	*c*, Å	*γ*, °	R_wp_, %	R_p_, %
SC	reflection	7.952(1)	8.167(4)	10.35(2)	96.3(1)	7.5	5.4
	transmission	7.952(2)	8.163(8)	10.36(1)	96.3(3)	9.4	7.6

**Table 4:** Calculation results for crystallinity index (CrI) and crystallite size (*D_hkl_*).

Geometry		Reflection	Transmission
CrI, %		81 ± 5	86 ± 5
[hkl]	(hkl)	Dhkl, Å (Cauchy-Gauss average)	
$1\bar{1}$0	$1\bar{1}$0	44 ± 5	43 ± 5
110	110	62 ± 5	63 ± 5
100	200	59 ± 5	58 ± 5
001	004	94 ± 5	93 ± 5

Note: ΔCrI = ±5%; Δ*D_hkl_* = ±5Å; [*hkl*]—crystallographic direction indices in cellulose Iβ; (*hkl*)—reflection indices on the X-ray diffractograms.

**Table 5:** Crystallinity index (CrI) and crystallite size (*D_hkl_*) for the SC-derived NC, calculated from the diffractogram in reflection.

Sample	CrI, %	Crystallite Size (*D_hkl_*, Å) for Crystallographic Plane Spacings (*d_hkl_*, Å) and Diffraction Peaks (2*θ*)			
SC-derived NC	17 ± 5	29 ± 5	62 ± 5	26 ± 5	38 ± 5
		7.38 ± 0.05	7.14 ± 0.05	4.82 ± 0.05	3.97 ± 0.05
		12.00	12.40	18.40	22.40

Note: ΔCrI = ±5%; Δ*D_hkl_* = ±5Å; Δ*d_hkl_* = ±0.05Å.

**Table 6:** Summary Table for NC from SC (DP of SC 3140).

Sample Name	Actual Yield, %	Nitrogen Content, %/DP	Viscosity of the 2% Solution in Acetone mPa·s	Solubility in Alcohol–Ether Solvent, %	Ash Content, %	FT-IR Spectroscopy, cm^−1^	TGA/DTA	CrI, %/Crystallite Sizes, Å	Morphology
							T_Onset_/T_Peak_, °C/Q, kJ/g		
NC from SC (SC DP of 3140)	138 ± 2	11.83 ± 0.05/2.21	198 ± 2	91 ± 2	0.05 ± 0.01	1644, 1278, 832, 746, 662	200/208/7.74	17 ± 5/29 × 62 × 26 × 38	NC fibers have a cylindrical shape with a diameter increasing to 25 µm; the surface is covered with numerous microcracks and roughness

Note: NC, cellulose nitrate; *T*_Onset_, onset temperature of intense decomposition of the sample; *T*_Peak_, peak temperature of intense decomposition of the sample; *Q*, specific heat of decomposition of the sample, kJ/g.

## Data Availability

The original contributions presented in this study are included in the article. Further inquiries can be directed to the corresponding author.
